# Serious Game with Electromyography Feedback and Physical Therapy in Young Children with Unilateral Spastic Cerebral Palsy and Equinus Gait: A Prospective Open-Label Study

**DOI:** 10.3390/s24051513

**Published:** 2024-02-26

**Authors:** Christophe Boulay, Jean-Michel Gracies, Lauren Garcia, Guillaume Authier, Alexis Ulian, Maud Pradines, Taian Martins Vieira, Talita Pinto, Marco Gazzoni, Béatrice Desnous, Bernard Parratte, Sébastien Pesenti

**Affiliations:** 1 Gait Laboratory, Pediatric Orthopaedic Surgery Department, Timone Children Hospital, 13385 Marseille, France; lauren.garcia0204@gmail.com (L.G.); guillaume.authier@ap-hm.fr (G.A.); alexis.ulian@ap-hm.fr (A.U.); bernard.parratte@gmail.com (B.P.); sebastien.pesenti@ap-hm.fr (S.P.); 2 Aix-Marseille University, CNRS, ISM UMR 7287, 13284 Marseille, France; 3 AP-HP, Service de Rééducation Neurolocomotrice, Unité de Neurorééducation, Hôpitaux Universitaires Henri Mondor, F-94010 Créteil, France; jean-michel.gracies@aphp.fr (J.-M.G.); maudprad@gmail.com (M.P.); 4 UR 7377 BIOTN, Laboratoire Analyse et Restauration du Mouvement, Université Paris Est Créteil (UPEC), F-94000 Créteil, France; talitappinto@gmail.com; 5 Laboratory for Engineering of the Neuromuscular System (LISiN), Department of Electronics and Telecommunication, Politecnico di Torino, 10129 Turin, Italy; taian.martins@polito.it (T.M.V.); marco.gazzoni@polito.it (M.G.); 6 PoliToBIOMed Laboratory, Department of Electronics and Telecommunications, Politecnico di Torino, Corso Duca degli Abruzzi 24, 10129 Turin, Italy; 7 Instituto D’Or de Pesquisa e Ensino (IDOR), Rio de Janeiro 22281-100, Brazil; 8 Pediatric Neurology Department, Timone Children Hospital, 13005 Marseille, France; beatrice.desnous@ap-hm.fr

**Keywords:** children with cerebral palsy, serious game, gait velocity, five-step assessment, coefficients of impairment, equinus gait

## Abstract

The clinical effects of a serious game with electromyography feedback (EMGs_SG) and physical therapy (PT) was investigated prospectively in children with unilateral spastic cerebral palsy (USCP). An additional aim was to better understand the influence of muscle shortening on function. Thirty children with USCP (age 7.6 ± 2.1 years) received four weeks of EMGs_SG sessions 2×/week including repetitive, active alternating training of dorsi- and plantar flexors in a seated position. In addition, each child received usual PT treatment ≤ 2×/week, involving plantar flexor stretching and command strengthening on dorsi- and plantar flexors. Five-Step Assessment parameters, including preferred gait velocity (normalized by height); plantar flexor extensibility (XV1); angle of catch (XV3); maximal active ankle dorsiflexion (XA); and derived coefficients of shortening, spasticity, and weakness for both soleus and gastrosoleus complex (GSC) were compared pre and post treatment (*t*-tests). Correlations were explored between the various coefficients and gait velocities at baseline. After four weeks of EMGs_SG + PT, there was an increase in normalized gait velocity from 0.72 ± 0.13 to 0.77 ± 0.13 m/s (*p* = 0.025, d = 0.43), a decrease in coefficients of shortening (soleus, 0.10 ± 0.07 pre vs. 0.07 ± 0.08 post, *p* = 0.004, d = 0.57; GSC 0.16 ± 0.08 vs. 0.13 ± 0.08, *p* = 0.003, d = 0.58), spasticity (soleus 0.14 ± 0.06 vs. 0.12 ± 0.07, *p* = 0.02, d = 0.46), and weakness (soleus 0.14 ± 0.07 vs. 0.11 ± 0.07, *p* = 0.005, d = 0.55). At baseline, normalized gait velocity correlated with the coefficient of GSC shortening (R = −0.43, *p* = 0.02). Four weeks of EMGs_SG and PT were associated with improved gait velocity and decreased plantar flexor shortening. A randomized controlled trial comparing EMGs_SG and conventional PT is needed.

## 1. Introduction

Infant paresis, also known as ‘cerebral palsy’ (CP), is a developmental neurological and muscular disorder due to early non-progressive brain injury, leading, among other consequences, to spinal cord development disorder [1,2]. Motor impairment limits activities, participation, and quality of life, particularly linked with decreased gait velocity [3,4]. The prevalence of CP has remained stable at around 2.5 per 1000 births [5] in western countries, with a trend towards a relative increase in unilateral disorders (unilateral spastic cerebral palsy, USCP) [6,7]. Children often present with equinus gait [8,9,10], which is attributed to reduced extensibility and overactivity of ankle plantar flexors together with weakness of command to dorsiflexors [11,12] (Figure S1).

A syndrome of deforming spastic paresis develops in USCP, which includes a muscle disorder, *spastic myopathy*, clinically manifested by muscle extensibility loss, and a neural disorder comprising overactivity in antagonists (spastic dystonia, spastic cocontraction, and spasticity) and weakness (stretch-sensitive paresis) in agonists [11,12,13,14]. The entanglement of the muscle and the neurological disorders leads to dysregulation between agonist and antagonist muscle activation.

The Five-Step Assessment (FSA) has been developed in this context as a stepwise method to *clinically quantify* spastic paresis using measurements of antagonist stretches and ranges of motion in degrees, based on the concept of resisting antagonists as the main cause of motor impairment [11,12,15,16,17,18]. From X_V1_ (*angle of arrest* upon slow and strong stretch of the tested antagonist, i.e., maximal clinical extensibility of the tested antagonist, examined at rest), X_V3_ (*angle of catch* of the antagonist upon fast stretch, examined at rest, i.e., threshold of the stretch reflex of the antagonist), and X_A_ (*angle of match* between agonist-induced torque and antagonist resistances, i.e., maximal range of active motion against the resistance of the antagonist), coefficients of impairment have been derived, including coefficients of shortening (C_SH_), spasticity (C_SP_), and weakness (C_w_) (see Section 2). These coefficients provide estimates of the respective contributions of the muscular and neural disorders in deforming spastic paresis [19,20,21]. The relevance of these coefficients has not been documented in children with USCP.

Physical therapy (PT) plays the central role in the treatment of motor impairment following stroke among adults [22,23,24] and children [25,26]. Early child active motor learning interventions appear to improve movement and cognition compared to passive approaches in children with CP [26]. Yet, the optimal techniques remain uncertain [27]. Novak et al. [26] have reported on the low quality of evidence for some of the interventions used, such as massage or stretching. Another common limitation of PT management when using long rehabilitation programs is that children drop out, with this treatment depending on children’s and parents’ availability and willingness to attend PT sessions.

Biofeedback strategies represent an area of current intervention and research in children with CP, although lower limb studies with EMG biofeedback have been scarce [26,28]. In the past 20 years, a wide range of game-oriented interventions has emerged for the rehabilitation of children with CP, widely known as serious games [29]. Gamifying EMG biofeedback rather than presenting it with conventional acoustic or visual means [30] aimed to increase the playfulness of interventions so as to keep children better motivated [29,31]. Motivation and attention are indeed vital modulators for rehabilitation compliance and neuroplasticity [26]. Although effective [32,33], commercially available serious games for children with CP have mainly focused on the use of movement data; none seemed to directly use a key source of motor impairment, i.e., inappropriate muscle activity, using EMG-based serious games (EMGs_SG) [11]. EMGs_SG may provide therapists with ongoing data on agonist and antagonist recruitment while using repetitive alternating movement training in particular [34,35].

In this study, we used supervised EMGs_SG in combination with in-hospital PT to control treatment adherence and progress while prompting repetitive alternating movement training in young children with USCP [36,37]. The hypothesis was that the combination of EMGs_SG and PT in USCP children might improve gait function by decreasing plantar flexor shortening and overactivity as measured by the FSA [30,38].

## 2. Materials and Methods

### 2.1. Study Design

This prospective interventional open-label study was approved by the national ethics committee (2018-A00831-54) and was conducted in compliance with the Declaration of Helsinki. All parents and children gave written informed consent before participation.

### 2.2. Study Subjects

Children with USCP under care at a university children’s hospital were prospectively screened between July 2019 and April 2021 for the following eligibility criteria: age 4–10, equinus gait pattern (equinovarus, equinovalgus), Level I or II on the Gross Motor Function Classification System (GMFCS) [39], and sufficient cognition to understand and play the EMGs_SG. Children were excluded if they had received surgery or focal administration of botulinum toxin within the past 6 months, showed severe gastrosoleus complex (GSC) contracture (maximum passive dorsiflexion, knee extended, X_V1-GSC_ < 70°, i.e., less than −20° of dorsiflexion), or presented with a leg length difference > 1 cm.

### 2.3. Intervention

All participants received EMGs_SG treatment sessions twice a week over four weeks. EMGs_SG treatment was applied by the same therapist, with each game session lasting 30 min. During the four weeks, children also participated in their usual PT outside of the neurorehabilitation center in community-based PT offices. PT treatment of equinus consisted of PT sessions once or twice a week, outside the EMGs_SG sessions days, comprising a physiotherapy program including plantar flexor stretching coupled with strengthening of tibialis anterior (TA) and plantar flexors.

**Figure 1:**
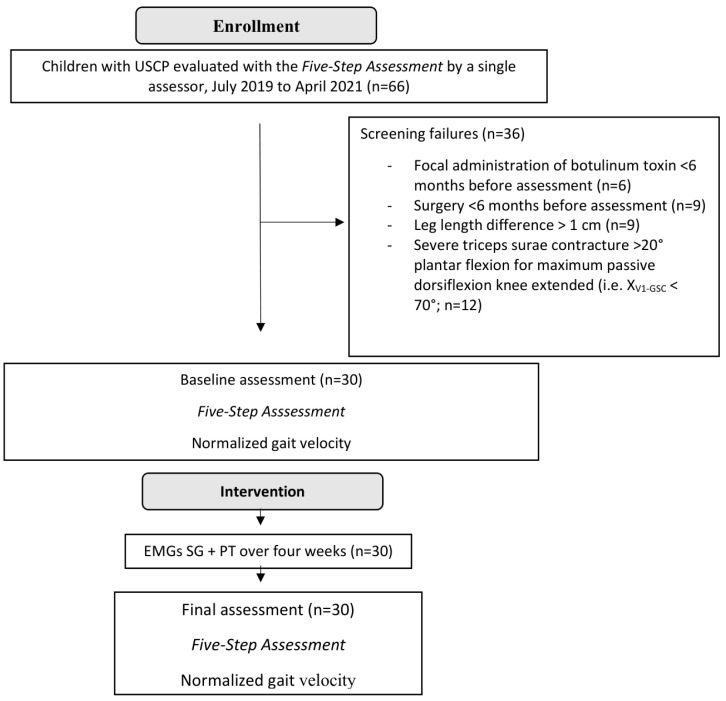
Flow diagram of patients throughout the course of the study.

All participants were evaluated at baseline and after the four weeks of EMG_SG and PT treatment (Figure 1). Evaluations were carried out by the same therapists with over 15 years of experience in the assessment of children with CP.

### 2.4. Serious Game Using EMG Biofeedback

The EMGs_SG by EMG biofeedback used here involves recording muscle activity in the paretic lower limb and transcribing it live on screen, offering children visual feedback on their muscle activities. EMGs_SG training is a platform game in which participants can control the movements of a game character modulated according to the amplitude of surface EMGs. EMGs amplitudes (microV) reflect motor unit recruitment. Surface EMG electrodes (12 mm diameter, Ambu, Ballerup, Denmark) were placed on TA and gastrocnemius medialis (GM) muscles after cleaning the skin (Nuprep, Norwalk, CA, USA) and were connected via Bluetooth to an amplifier (DuePro, OT Bioelettronica in Italy). Children played in seated position, knee flexed, and were requested to move a game character (bee or helicopter) up and down to hit as many targets (flowers or stars) as possible by activating dorsiflexors or plantar flexors (see EMGs_SG video applications in the Supplementary Materials). Feedback was delivered through a combination of audio and visual rewards during the entire game depending on TA and GM electromyographic activities. EMGs_SG focused the attention on the performance correlated with the proportion to the targets reached (flowers or stars) [28]. The difficulty level and EMG feedback were calibrated for each child to their maximum voluntary contraction of each muscle (TA/GM), measured in the knee flexed position before each session. Difficulty was kept constant across sessions by varying the target position from 15% to 95% of the screen height, depending on the performance of each participant. Five minutes of familiarization were provided before the start of the EMGs_SG session. Each EMGs_SG session (30 min) randomly involved 10 min of required ankle dorsiflexor activation (agonists), 10 min of required plantar flexor activation (antagonists), and 10 min of required alternating dorsi- and plantar flexor activation.

### 2.5. Outcome Measure-Quantified Clinical Evaluation

Clinical assessment used the first three technical steps of the Five-Step Assessment (FSA) [16] involving angle measurements of X_V1_, X_V3_, and X_A_ for soleus and GSC muscles. Measurements utilized a goniometer with the participant in a relaxed supine position, knee flexed to assess soleus and knee extended to assess GSC. The range of passive ankle dorsiflexion (angle of arrest X_V1_) was measured using the slowest and strongest possible stretch to move the ankle segment as far up as possible, which provided information on the maximal clinical extensibility of primarily the muscle tissue [40]. X_V3_, the angle of catch, was measured by applying the fastest possible plantar flexor stretch, providing information on the stretch reflex threshold. X_A_, the maximal range of active ankle dorsiflexion, was the angle of match between maximal agonist effort and associated antagonist resistances (active through cocontraction and passive).

### 2.6. Coefficients of Impairment

Three coefficients of impairment were derived from the X_V1_, X_V3_, and X_A_ parameters for each of soleus and GSC.

- The coefficient of shortening (C_SH_) is derived from X_V1_ based on the formula C_SH_ = (X_N_ − X_V1_)/X_N_. X_N_ is the maximal expected physiological range for the joint considered, here defined according to normative values of typically developed children of a similar age range [41]. In this study, for children aged between 4 and 7, the normal reference X_N_ for the ankle dorsiflexion was considered to be 119° knee flexed and 113° knee extended, while for children between 8 and 11, it was considered to be 117° knee flexed and 112° knee extended [41].
- The coefficient of spasticity (C_SP_) is defined by the ratio C_SP_ = (X_V1_ − X_V3_)/X_V1._ C_SP_ evaluates spasticity, taking into account the maximal clinical extensibility of the tested muscle.
- The coefficient of weakness (C_w_) is defined by the ratio C_W_ = (X_V1_ − X_A_)/X_V1_ to estimate the impairment of active command, once taking maximal passive extensibility of the antagonist into account [19]. The intra- and inter-rater reliabilities of C_SH_, C_SP_, and C_w_ have been previously demonstrated [42].

### 2.7. Normalized Gait Velocity

Gait velocity was assessed using an eight-camera system (100 Hz, MxT40, Vicon, Oxford, UK) and a set of 16 reflective markers according to the adapted Plug-in-Gait model. Children walked barefoot over a 10 m walkway at their preferred speed, until 3 to 6 gait cycles were collected. Gait velocity measurements were determined for a representative gait cycle [43]. Normalized gait velocity was calculated to take the subject height into account [44].

### 2.8. Statistical Analysis

Shapiro–Wilk tests were used to test for normal distribution of data (*p* > 0.05). Greenhouse–Geisser estimates of sphericity were used to correct degrees of freedom wherever Mauchly’s test was significant. *t*-tests for paired data were used to compare the clinical parameters (X_V1_, X_A_, and X_V3_), coefficients of impairments, and normalized gait velocity pre and post EMGs_SG + PT. Univariable regression analyses were carried out to explore correlations between raw values (X_V1_, X_V3_, and X_A_), coefficients of impairments, and the normalized gait velocity at baseline. In the two subgroups below or above the median of coefficient of shortening pre EMGs_SG + PT, correlations between coefficients of shortening and spasticity were explored. Effect sizes were calculated. Significance was set at *p* < 0.05. All analyses were performed using Statistical Jasp 0.16.0.0 version software (Flower Mound, TX, USA).

## 3. Results

### 3.1. Patients

From the 66 screened children with USCP, 30 met the eligibility criteria. The reasons for ineligibility are provided in the flow chart of patient recruitment (Figure 1). Clinical characteristics before EMGs_SG intervention are reported in Table 1.

### 3.2. Changes in Clinical and Functional Performances

**Figure 2:**
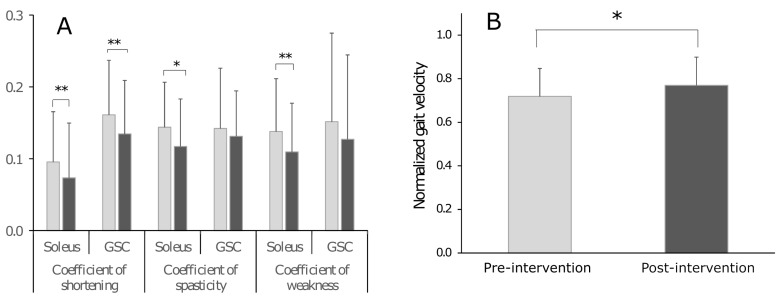
Effects of the intervention (EMGs_SG and PT) on clinical assessment (**A**) and on gait velocity (**B**). * *p* < 0.05; ** *p* < 0.01. EMGs_SG and PT, serious game and physical therapy.

There was a decrease in the coefficient of shortening post EMGs_SG and PT for both soleus (0.10 ± 0.07 vs. 0.07 ± 0.08, *p* = 0.004, d = 0.57, pre vs. post) and GSC (0.16 ± 0.08 vs. 0.13 ± 0.08, *p* = 0.003, d = 0.58) (Figure 2A, Table 2). The coefficients of spasticity (0.14 ± 0.06 vs. 0.12 ± 0.07, *p* = 0.018, d = 0.46) and weakness (0.14 ± 0.07 vs. 0.11 ± 0.07, *p* = 0.005, d = 0.55) also decreased for the soleus muscle, whereas no change in the coefficients of spasticity and weakness were observed for GSC (Table 2). Normalized gait velocity increased by 7% (0.72 ± 0.13 vs. 0.77 ± 0.13, *p* = 0.025, d = 0.43) (Figure 2B, Table 2).

### 3.3. Relationships between Technical Parameters and Normalized Gait Velocity

**Figure 3:**
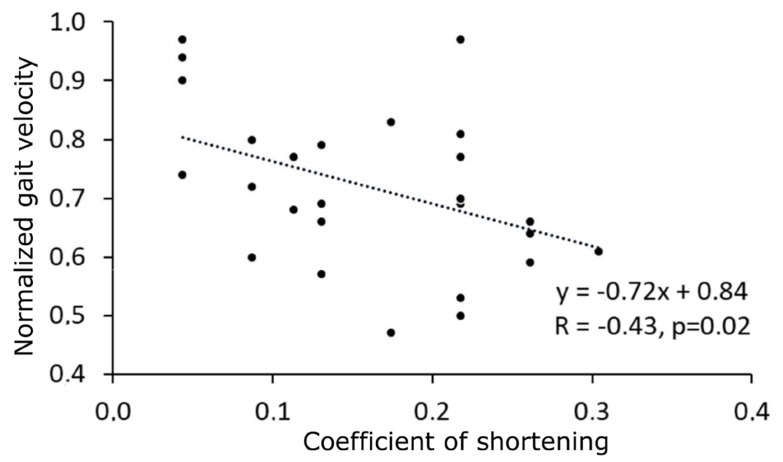
Baseline relationship between GSC coefficient of shortening and normalized gait velocity. GSC, gastrosoleus complex.

At baseline, normalized gait velocity correlated with X_V1_ of GSC and with the coefficient of shortening of GSC (Figure 3).

### 3.4. Relationships between Changes in Coefficients

**Figure 4:**
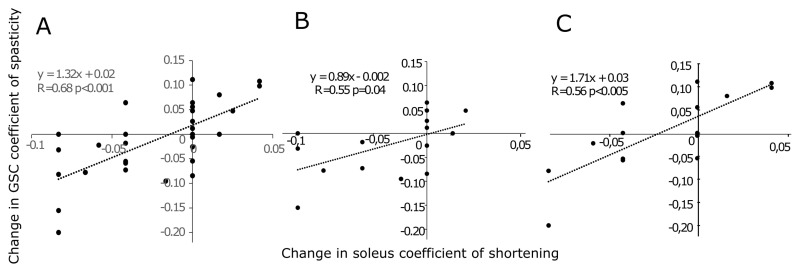
Relationship between changes in soleus coefficient of shortening coefficient (post minus pre-intervention) and changes in GSC coefficient of spasticity (post minus pre-intervention) for the entire sample (**A**) for children with a baseline soleus coefficient of shortening below the median (≤0.08) (**B**) and for children with a baseline soleus coefficient of shortening above the median (>0.08) (**C**). GSC, gastrosoleus complex.

The changes in the coefficient of spasticity of GSC correlated with the changes in the coefficient of shortening of soleus (Figure 4A), a correlation that was stronger in the group with a less extensible soleus at baseline (Figure 4B,C).

## 4. Discussion

This prospective open-label study evaluated the effects of four weeks of two weekly sessions of an EMG-biofeedback-based serious game involving ankle muscle training practiced in seated position combined with PT in children with USCP. We observed positive functional and technical effects, with an increase in normalized gait speed and a decrease in coefficients of shortening, spasticity, and weakness of the soleus as well as a decrease in the coefficient of shortening of GSC. In addition, correlations were shown between soleus lengthening through the four weeks of training and spasticity reduction in GSC.

### 4.1. Impact of EMGs_SG and PT on Normalized Gait Velocity

EMGs_SG intervention was adapted to the functional and cognitive performance of each child to maintain a greater level of concentration and to minimize fatigability. In this open-label study of EMGs_SG and PT, we observed an increase in normalized gait velocity (Figure 2B) reflecting functional improvement, which corroborates prior non-randomized findings by Dursun and colleagues after EMG biofeedback treatment [45].

### 4.2. EMGs_SG and PT Impact on Muscular and Neurological Disorders

The FSA, an expansion of the Tardieu scale, is a recent tool for quantified clinical measurements in spastic paresis that attempts to clinically estimate each of the muscle and the neurological disorders using specific coefficients of impairment [18,19]. The present study is the first to use these coefficients of impairment in CP children, which represents a novel method to evaluate treatment. Of note, participants in this study constitute a representative sample of children with USCP, i.e., presenting with typical proportions of GFMCS I and II (Table 1) [46]. Coefficients of impairment, particularly the coefficients of shortening [19], provided normalized clinical measurements from the FSA with respect to typical development [41]. In this prospective study, we observed decreased soleus and GSC shortening as well as decreased soleus spasticity and increased active dorsiflexion knee flexed (Table 2). Effect sizes for soleus and GSC X_A_ improvements were strong (d > 0.80), likely reflecting meaningful balance improvement between agonists and antagonists during active dorsiflexion efforts. Coefficients of impairment, derived from the raw measurements of FSA, for soleus showed the same results as the raw X_V1_, X_V3_, and X_A_ (Table 2, Figure 2A), in contrast with the GSC results, for which the coefficients of spasticity and of weakness did not significantly decrease (Table 2, Figure 2A). This might have to do with the position in which children trained, i.e., the seated position, knee flexed, a position in which gastrocnemius muscles were not under stretch. This may thus reflect selectivity of the effects of muscle training, as well as the importance of stretch to improve active command parameters.

The improved active dorsiflexion ranges after treatment suggest balance improvement between agonists and antagonists: repetitive and particularly *alternating* active dorsiflexion training may lead to decreasing active (spastic cocontraction) and passive resistance (reflected by X_V1_ or shortening, together with X_V3_ or spasticity) from plantar flexors, as has been demonstrated in adults after long-term guided self-stretch and active training [47,48]. Such improved regulation between agonists and antagonists after training in alternating efforts has been previously suggested in spastic paresis and may involve restored reciprocal inhibition, as is known from healthy subjects [47,49,50].

### 4.3. Impact of Lengthening Soleus on the Command of Active Dorsiflexion

The syndrome of deforming spastic paresis in USCP comprises a muscle and a neurological disorder. The muscle disorder (spastic myopathy) involves a loss of extensibility of the muscle–tendon complex [11,12,13,20,51,52,53,54,55,56,57]. The neurological disorder comprises overactivity in antagonists (spastic cocontraction, spastic dystonia and spasticity) and agonist paresis [11,12,13,14,58]. In chronic acquired hemiparesis in adults, past a threshold of severity, the muscle disorder may contribute to worsen the neurological disorder by further altering the descending command [20]. Among very young USCP children, the present study may corroborate these findings [20], as the reduction of gastrocnemius spasticity correlates with the decrease in soleus shortening. In the present study, a tipping point may exist where such a relationship is strengthened (Figure 4). Spasticity has been defined as an increase in the velocity-dependent reflexes to phasic stretch, detected and measured at rest [13]. It is known that such stretch reflex hypersensitivity is enhanced when the muscle–tendon complex is contractured, as spindle firing is then enhanced for a given muscle stretch [11,12,20,59,60,61]. The heteronymous relationship between the decreases in soleus shortening and in gastrocnemius spasticity may involve reduced afferent soleus activity due to decreased intramuscular tension in a lengthened soleus muscle (through the present therapeutic intervention), with a then lesser degree of heteronymous facilitation from soleus afferents to gastrocnemius motor neurons, which might contribute to their decreased spasticity [62,63].

### 4.4. The Role of Muscle Shortening in Limiting Gait Velocity

Similarly to adults presenting with acquired chronic hemiparesis, GSC shortening seemed to significantly impact gait velocity among our sample (Figure 3). Thus, GSC shortening conditions normalized gait velocity in pre EMGs_PT (Figure 3), which is not confirmed post EMGs_PT. This is an argument for the deleterious role of GSC shortening and the value to treat muscle shortening in CP children. Our findings suggest the key role of muscular–aponeurosis complex extensibility on functional performance in children with USCP: we would encourage working on the feasibility of intensive, dynamic, active stretching programs for GSC in the early treatment of paretic children [64,65].

### 4.5. Study Limitations

This was an open-label, short-term study on a limited number of children. It would have been interesting to monitor ankle *movements* and not just EMG, perhaps even muscle tension during the sessions, thus quantifying the amount of actual muscle stretch obtained through the sessions. Another limitation is the specific EMGs_SG application used here: EMGs_SG was performed with children seated knee flexed and thus in a semi-closed kinetic chain with no stretch imposed on GSC. This may explain the lesser results on GSC. EMGs_SG application in the *standing* position could have led to children’s fatigability and dropouts, but this remains to be tested. An additional limitation is the assessment methodology: the clinical measurements of X_V1_ mainly assessed passive extensibility, but residual spastic dystonia cannot be completely eliminated (neurological disorder) [20]. Finally, evaluation of the perceived effort in children could have been proposed, as in the Children’s Effort Rating Table [66]. Motivation and satisfaction could have been measured [67].

## 5. Conclusions

Our experience with EMGs_SG is that it is an innovative and an amusing way to improve clinical outcomes and gait velocity in children with an equinus gait. In this group of patients, EMGs_SG and PT were associated with improvements in ICF (International Classification of Functioning, Disability and Health) Activities and Participation and Body Function domains. EMGs_SG allows children to be the agents of their own treatment and may thus increase their adherence to their rehabilitation program and their motivation. This coaching-based approach could be considered as a home-based rehabilitation program with EMGs_SG carried out by parents.

Our study provides additional evidence on the benefits of early EMGs biofeedback and PT in children with USCP [26,28,68]. EMGs_SG can also be adapted to the functional and cognitive performance of each child to maintain concentration levels and avoid fatigue. Here, difficulty levels were adjusted for each child in accordance with their functional performance based on calibration measurements before each session.

Our results support previous evidence of a probable link between repetitive active and alternative training and improved balance between agonists and antagonists [34,69,70], probably mediated by better regulated activation between those muscles [14,71,72,73,74,75]. Our findings also demonstrate the key impact of muscular–aponeurosis complex shortening (muscular aspect of the disorder) on neurological features (cocontraction, spasticity, dystonia, and paresis).

In very young children, this study emphasizes the need to focus on muscle shortening treatment by applying dynamic and active stretching programs on antagonists and by activated descending command with active training.

Our findings also suggest that EMGs_SG is more efficient than a standard physical therapy care in children with USCP, but further studies are required to confirm these initial findings, including randomized controlled trials comparing the outcomes of EMGs_SG and standard PT, as well as studies of the impact of botulinum toxin injection and of the importance of the standing position in the serious game in this context.

## Figures and Tables

**Table 1:** Clinical characteristics.

	Hemiparetic Children (*n* = 30)
Age (years)	data
Sex	7.6 ± 2.0
Weight (kg)	17 M/13 F
Height (m)	26.4 ± 7.1
BMI (kg/m^2^)	1.25 ± 0.12
Paretic side	16.6 ± 2.1
Gait velocity (m/s)	18 R/12 L
Normalized gait velocity	0.89 ± 0.15

Values expressed as mean ± SD.

**Table 2:** Clinical outcomes.

	Soleus (Knee Flexed)				Gastrosoleus Complex (Knee Extended)			
	Pre EMGs_SG + PT	Post EMGs_SG + PT	*p*	Effect Size	Pre EMGs_SG + PT	Post EMGs_SG + PT	*p*	Effect Size
**X_V1_ (°)**	108.5 ± 8.4	111.2 ± 9.2	0.004	0.58	96.5 ± 8.7	99.5 ± 8.6	0.002	0.61
**X_V3_ (°)**	93.0 ± 10.6	98.4 ± 12.5	<0.001	0.74	82.5 ± 9.0	86.5 ± 9.9	0.006	0.54
**X_A_ (°)**	93.6 ± 11.3	99.0 ± 10.6	<0.001	0.90	81.6 ± 12.8	86.8 ± 12.9	<0.001	0.83
**C_SH_**	0.10 ± 0.07	0.07 ± 0.08	0.004	0.57	0.16 ± 0.08	0.13 ± 0.08	0.003	0.583
**C_SP_**	0.14 ± 0.06	0.12 ± 0.07	0.018	0.46	0.14 ± 0.09	0.13 ± 0.06	0.417	0.150
**C_W_**	0.14 ± 0.07	0.11 ± 0.07	0.005	0.55	0.15 ± 0.13	0.13 ± 0.12	0.073	0.340
	**Normalized gait velocity**							
	0.72 ± 0.13	0.77 ± 0.13	0.025	0.43				

C_SH_, coefficient of shortening; C_SP_, coefficient of spasticity; C_W_, coefficient of weakness; EMGs_SG and PT, serious game and physical therapy; X_V1_, maximal passive range of motion against the resistance of the investigated muscle; X_V3_, angle of catch by applying stretch of the investigated muscle at the fastest possible velocity for the examiner; X_A_, angle of match between agonist-induced torque and antagonistic resistances, i.e., active range of motion against the resistance of the investigated muscle. Normalized gait velocity was calculated to take the subject height into account.

## Data Availability

Study data are available from the corresponding author on reasonable request.

## Supplementary Materials

The following supporting information can be downloaded at https://www.mdpi.com/article/10.3390/s24051513/s1 Figure S1: Illustration of neuro-orthopedic deformities in children with unilateral spastic cerebral palsy (USCP) and equinus gait. Figure S2: Change in shortening coefficient vs. normalized gait velocity. Video S1: Active alternative training. Video S2: Dorsiflexion active training. Video S3: Plantarflexion active training.
