# Supplementary material for: Serious Game with Electromyography Feedback and Physical Therapy in Young Children with Unilateral Spastic Cerebral Palsy and Equinus Gait: A Prospective Open-Label Study
Source: Sensors (Basel). 2024 Feb 26;24(5):1513. doi: 10.3390/s24051513 (PMC10935430; doi:10.3390/s24051513)
Supplement: Supplementary file 1 [file sensors-24-01513-s001.zip › Supplementary_material.pdf]

Appendix A. Supplemental data

Supplemental data associated with this article can be found, in the online version, at

*__Clinical measurements validity and reliability__ (X_{V1}, X_{V3} and X_A)

doi:10.1016/j.jelekin.2022.102665.

* **Relationships between technical parameters and normalized gait velocity**

There was a correlation between normalized gait velocity at baseline and the changes in the coefficient of shortening of soleus post treatment (Suppl Figure A1).

Interestingly, baseline normalized gait velocity pre EMGs_SG and PT was found to be a predictor of improvement in the coefficient of shortening of soleus in the present study (supplemental Figure A1). It is possible that incremental gains in soleus extensibility might have been better maintained in faster walking children, who will likely use greater number of larger steps (repeated minute plantar flexor stretches) every day.

* **The physical therapy part in the EMGs_SG and PT intervention**

The impact of PT could not be disentangled from that of EMGs_SG in the present study. PT could comprise a wide range of manual techniques and assisting devices depending on the clinician care and experience [25]. These sessions are likely to have had some effectiveness [27,28]. In real life however, appointments and follow up in community-based PT offices may become difficult to maintain in the long term, leading to rehabilitation dropouts. Therefore, EMGs_SG could constitute a novel, easy-to-use tool as a complementary technique to facilitate compliance among children.

*__Perspectives__

EMGs_SG represents a novel therapy and a fun way for children with USCP and equinus gait to work on ankle muscles and command. EMGs_SG allowed children to play an active role in their own treatment, akin to the situation in Guided Self-rehabilitation Contracts in adults [52]. EMGs_SG could promote children motivation and adherence to rehabilitation programs, allowing interactive and dynamic way of PT coaching. This coaching-based approach could be envisioned in home-based rehabilitation programs with EMGs_SG carried out by the parents. Finally, the present findings further suggest the role of changes in the muscle disorder in improving command, that is the neurological disorder.

***EMGs_SG videos applications** :

*dorsiflexion active training

*plantarflexion active training

*active alternative training

***Figure S1.** Illustration of neuro-orthopedic deformities in children with unilateral spastic cerebral palsy (USCP) and equinus gait. These patients have a combination of muscular and neurological disorders. The muscular disorder consists of a shortening of ankle plantar flexor muscles (antagonists). The neurological disorders include paresis of ankle dorsiflexors (agonists), especially the tibialis anterior, overactivity (dystonia, cocontractions, and spasticity) of plantar flexors (antagonists), especially the peroneus longus and triceps surae, and dysregulation between agonist and antagonist muscles.

***Figure S2.** Relationship between changes in soleus coefficient (post minus pre intervention) and normalized gait velocity at baseline.
