# Supplementary figures and images for: Serious Game with Electromyography Feedback and Physical Therapy in Young Children with Unilateral Spastic Cerebral Palsy and Equinus Gait: A Prospective Open-Label Study
Source: Sensors (Basel). 2024 Feb 26;24(5):1513. doi: 10.3390/s24051513 (PMC10935430; doi:10.3390/s24051513)

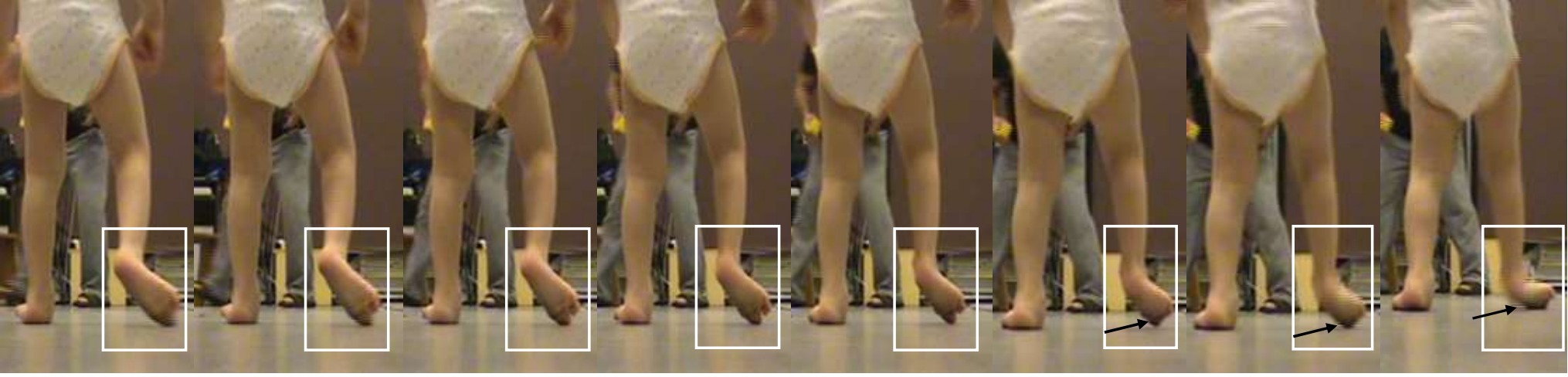

Supplement: Supplementary file 1 [file sensors-24-01513-s001.zip › Figure S1.pdf]

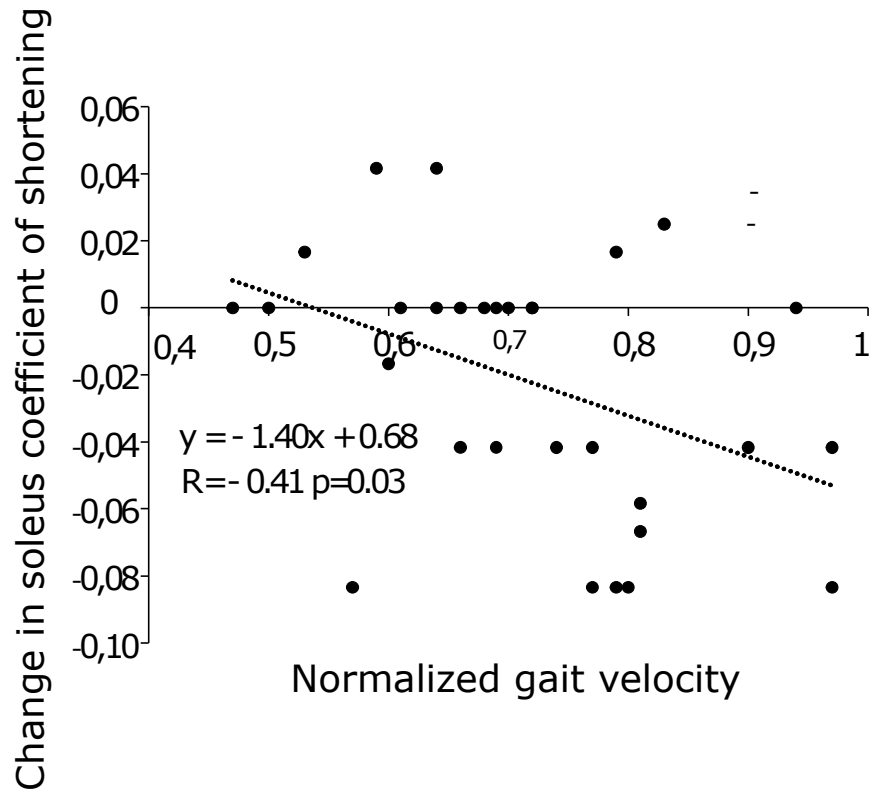

Supplement: Supplementary file 1 [file sensors-24-01513-s001.zip › Figure S2.pdf]
